# Quality of Life and Symptom Management in Advanced Biliary Tract Cancers

**DOI:** 10.3390/cancers13205074

**Published:** 2021-10-11

**Authors:** Lindsay A. Hunter, Heloisa P. Soares

**Affiliations:** Huntsman Cancer Institute, University of Utah, Salt Lake City, UT 84112, USA; heloisa.soares@hci.utah.edu

**Keywords:** biliary tract cancer, symptom management, palliative care, quality of life, gallbladder carcinoma, cholangiocarcinoma

## Abstract

**Simple Summary:**

Patients with advanced BTC have poor prognosis and frequently experience symptoms that adversely impact their quality of life. In this review, we explore the potential complications of advanced BTC and its treatments. We also review the possible strategies and interventions available to manage these adverse events.

**Abstract:**

Biliary tract carcinomas (BTCs) account for less than 1% of all cancers but are increasing in incidence. Prognosis is poor for BTC patients, with 5-year survival rates of less than 10%. While chemotherapy has been the mainstay treatment for patients with advanced BTC, immunotherapy and targeted therapies are being evaluated in numerous clinical trials and rapidly incorporated into clinical practice. As patients with BTC have reduced health-related quality of life (HRQoL) due to both tumor- and treatment-related symptoms, it is important for clinicians to recognize and manage these symptoms early. This review will highlight the anticipated complications from BTC and its systemic treatment, as well as their effects on HRQoL.

## 1. Introduction

Biliary tract carcinomas (BTCs) are a heterogeneous group of malignancies arising from the epithelial lining of the bile ducts or gallbladder [1,2]. While relatively uncommon in the United States, approximately 23,000 new cases diagnosed annually, the incidence of BTCs has been increasing [3,4,5]. Cholangiocarcinoma alone represents an estimated 3% of all gastrointestinal malignancies and is the second most common primary hepatic malignancy after hepatocellular carcinoma [5].

When diagnosing BTC, it is important to distinguish between intrahepatic cholangiocarcinoma (iCCA), extrahepatic cholangiocarcnoma (eCCA), gallbladder cancer (GBC), and ampulla of Vater carcinoma (AVC) as each sub-type has its own specific characteristics and variations in tumor biology [2]. Pathologic diagnosis is essential prior to any non-surgical treatment, but biopsies are often technically difficult or result in inadequate tissue sampling [6]. ERCP-guided biopsies are preferred, but EUS-guided fine needle aspiration may be considered if ERCP is unsuccessful. However, liquid biopsy utilizing ctDNA or cfDNA is gaining much attention as it may overcome many of the challenges inherent in diagnosing BTC [7].

Prognosis is typically dismal for patients with BTC. Five-year survival rates are currently less than 5% for unresectable and less than 40% for resectable tumors [1,8]. As BTCs are generally asymptomatic in early stages, 60–85% of patients present with metastatic or unresectable disease [6,8]. Even in the 30–40% of patients with resectable disease who undergo potentially curative surgery, approximately 50% develop recurrent disease [9,10,11]. Although 5-year overall survival (OS) following curative surgical resection differs based on anatomic site, for patients with cholangiocarcinoma (CCA), it is estimated to be 40% in radical (R0) resection but falls to 20% in cases with nodal and vascular involvement [11].

Systemic chemotherapy is the standard treatment for advanced BTC and can currently lead to a median OS of approximately 12 months [6]. Immunotherapy and targeted therapies are also emerging as a promising treatment options for advanced BTC. A number of actionable alterations have been or are currently under investigation in clinical trials in both the front-line and chemotherapy-refractory setting [6,12].

Health-related quality of life (HRQoL) has been found to be prognostic of survival in patients with hepatobiliary cancers even when controlling for disease-specific factors, treatment-related factors and demographics [13,14]. Unfortunately, advanced BTCs are associated with poor HRQoL at baseline [15,16,17,18]. Multiple symptom burden is common in patients with BTCs and often results in the rapid decline of physical functioning and emotional well-being [18,19]. While some physical symptoms are common to patients with advanced cancer, many are unique to BTC and its treatment. The aim of this review is to draw attention to tumor- and treatment-related complications unique to patients with BTC.

## 2. Local and Systemic Consequences of Advanced Disease

Patients with BTC often experience symptoms from both systemic and local consequences of disease. Frequently reported symptoms include jaundice, abdominal pain, pruritus, nausea, unintentional weight loss, fever, and fatigue [20,21,22]. In addition to directly impacting QoL, these symptoms have also been found to adversely impact emotional well-being, as well as physical and cognitive functioning [15,16]. Early and effective symptom management has the potential to improve quality of life, as well as mortality, for many patients.

Patients with advanced BTC have a particularly high chance of developing obstructive complications. Gastric outlet obstruction may be amenable to endoscopic duodenal stent placement. As patients often can only consume clear or full liquid diet post-procedure, duodenal stent placement may be a better option for palliation in patients with poor performance status, significant comorbidities and/or limited life expectancy [23,24]. Pancreatic duct obstruction may lead to exocrine pancreatic insufficiency, which can be managed with pancreatic enzyme replacement therapy [24].

Over 90% of patients with extrahepatic biliary cancer present with jaundice due to biliary obstruction [20]. Although a less common presenting symptom, acute cholangitis due to malignant biliary obstruction can be life-threatening. Adequate biliary drainage is critical not only in managing acute cholangitis and symptoms related to jaundice, such as pruritis, anorexia, and sleep disturbances, but also in enabling palliative systemic treatment. ERCP-guided biliary stent placement is the preferred strategy for palliating malignant biliary obstruction, especially in patients with poor performance status. However, percutaneous biliary draining may be pursued in patients where ERCP has been unsuccessful [25].

Although ERCP is generally felt to be a safe procedure, there are several serious complications to be aware of including acute cholangitis. In one retrospective review, more than 20% of patients with malignant hilar biliary obstruction experienced post-ERCP cholangitis [26]. Higher rates of post-ERCP cholangitis in patients with malignant hilar biliary obstruction are likely due to difficulties in achieving complete drainage in this population [26,27]. Stent type has also been identified as a possible risk factor for post-ERCP cholangitis. Several prospective and retrospective studies comparing metal with plastic stents found numerically lower rates of post-ERCP cholangitis with metal stents [26,28,29]. It should be noted that plastic biliary stents require replacement approximately every 3 months due to relatively high occlusion rate. Consequently, metallic biliary stents, which remain patent on average 7 months, may be preferred in many patients with advanced BTC [23,25].

## 3. Systemic Chemotherapy: Adverse Effects and Impact on Quality of Life

Chemotherapy may relieve tumor-related symptoms, improve quality of life, and prolong survival in select patients with advanced BTC [30]. In others, particularly patients with already poor performance status or very advanced disease, systemic chemotherapy can lead to a rapid decline in HRQoL [16,18]. A clear understanding of how systemic chemotherapy impacts quality of life is vital in determining which patients benefit from treatment and in navigating treatment discussions.

The phase III ABC-02 trial established the combination of gemcitabine and cisplatin as the standard of care first-line treatment for patients with advanced BTC [31]. Eligible patients with nonresectable, recurrent or metastatic BTC (iCCA, eCCA, GBC, or AVC) were randomly assigned to receive gemcitabine plus cisplatin or gemcitabine alone for up to 24 weeks. Randomization was stratified by primary tumor site, extent of disease, recruiting center, previous therapy, and performance status. Of the total 410 patients enrolled, 58.7% were reported to have a primary tumor arising from the bile duct. The median survival was statistically longer at 11.7 months in the gemcitabine plus cisplatin group compared to 8.1 months in the gemcitabine group (*p* < 0.001). These results were then supported by the Japanese phase II BT22 study which also compared this combination to gemcitabine alone in patients with locally advanced or metastatic BTC [32].

The combination of gemcitabine plus cisplatin has a favorable toxicity profile even when compared to gemcitabine. ABC-02 reported similar frequencies of grade 3 or 4 adverse effects between gemcitabine plus cisplatin (70.7%) and gemcitabine alone (68.8%) [31]. The number of patients who discontinued treatment due to toxicity was also similar between gemcitabine plus cisplatin and gemcitabine groups (10.4% versus 8.6%). Notable grade 3 or 4 toxic effects of gemcitabine plus cisplatin included neutropenia (25.3%), anemia (7.6%), thrombocytopenia (8.6%), fatigue (18.7%), nausea (4.0%), and vomiting (5.1%). Grade 3 or 4 abnormal liver function was also seen but was higher in the gemcitabine alone group (27.1% versus 16.7%), likely due to poorer local disease control.

BT22 reported a similar toxicity profile with the most common grade 3 or 4 adverse events being neutropenia (56.1%), thrombocytoepnia (39.0%), and anemia (36.6%) [32]. Common adverse events of any grade included anorexia (80.5%), nausea (68.3%), fatigue (58.5%), vomiting (48.8%), constipation (36.6%), and diarrhea (31.7%). Within the gemcitabine plus cisplatin arm, a total of 17% of patients discontinued treatment due to adverse events, and 9.7% of patients required dose adjustments.

Rates of treatment compliance and adverse events reported in clinical trials do not necessarily translate into how patients tolerate treatment in practice. To this end, the long-term outcomes and quality of life data of patients enrolled in ABC-02 was published in 2016 [14]. A total of 324 patients consented to completing the EORTC QLQ-C30 HRQoL questionnaire, with only 268 (83%) patients returning at least one form. Treatment mean differences in the HRQoL at 12 weeks between combination therapy and gemcitabine was not statistically significant for the majority of scales assessed. After controlling for patient characteristics and baseline HRQoL, only digestive symptoms and appetite loss were statistically significant at the 1% level, both in favor of gemcitabine plus cisplatin. While 59% of the data collected was missing at 12 weeks due to patient illness or death, the data is suggestive that HRQoL was not adversely affected.

Other gemcitabine-based or fluoropyrimidine-based regimens have demonstrated activity in clinical trials and are felt to be appropriate alternatives in the first line setting to gemcitabine plus cisplatin [33,34,35]. In select cases of locally advanced, unresectable BTC, particularly CCA, chemoradiation may also be considered as it can provide local symptom control an may potentially prolong OS [36]. Meta-analysis of BT22 and ABC-02 found patients with poorer performance status, ECOG 2, appeared to derive the least benefit from combination chemotherapy [35]. Therefore, in the absence of studies specifically for patients with poorer performance status, these findings suggest that monotherapy should be preferred in this population if chemotherapy is being considered at all and highlight the importance of early discussions focused on goals of care.

Due to the aggressive behavior of BTC and rapid decline of HRQoL associated with very advanced disease, further chemotherapy after failure of first line treatment is often challenging and/or contraindicated. Recently, the phase III ABC-06 trial determined the addition of FOLFOX to active symptom control (ACS) improved median OS in patients with advanced BTC (iCCA, eCCA, GBC, or AVC) after progression on gemcitabine and cisplatin compared to ASC alone (6.2 months versus 5.3 months) [37]. With regard to primary tumor site, iCCA was the most common site reported (44.4%), followed by eCCA (27.8%) and GBC (21.0%). Notably, patients enrolled in ABC-06 were particularly good candidates for further chemotherapy as eligibility criteria included an ECOG performance status of 0-1 and a life expectancy greater than 3 months. With regard to toxicity, grade 3 to 5 adverse events were reported in 69% of patients in the ASC plus FOLFOX group compared to 52% in the ASC alone group. The most commonly reported grade 3 to 5 chemotherapy-related adverse events were neutropenia (12%), fatigue or lethargy (11%), and infection (10%). The ASC plus FOLFOX group also experienced high frequency of grade 1 or 2 neuropathy (64%), nausea (37%), oral mucositis (35%), anorexia (31%), diarrhea (27%), thrombocytopenia (22%), and dysgeusia (20%).

Similarly, the phase IIB NIFTY trial presented at the American Society of Clinical Oncology (ASCO) 2021 found the addition of liposomal irinotecan (nal-IRI) to fluorouracil (5-FU)/leucovorin (LV) improved progression free survival (PFS) and OS compared to 5-FU/LV alone in patients with metastatic BTC after progression on gemcitabine plus cisplatin [38]. Patients with histologically or cytologically confirmed iCCA, eCCA, or GBC were enrolled and stratified by tumor site, as well as previous curative-intent surgery, prior to randomization. Patients with iCCA made up 42.5% of the study population. Ultimately, the median PFS was 7.1 months compared to 1.4 months, and the median OS was 8.6 months compared to 5.5 months, for nal-IRI plus 5-FU/LV and 5-FU/LV, respectively. The study was conducted in Korea only; otherwise, the eligibility criteria selected for a population similar to ABC-06 as ECOG 0-1 was required. Grade 3 to 5 adverse events were reported in 77.3% of patients in the nal-IRI plus 5-FU/LV arm compared to 31.4% of patients in the 5-FU/LV arm. The nal-IRI plus 5-FU/LV group experienced higher frequency of neutropenia (33.0%), fatigue (30.7%), constipation (29.5%), diminished appetite (27.3%), and nausea (25.0%) of any grade.

As the median OS benefit seen in both ABC-06 and NIFTY is modest, HRQoL data is pivotal to evaluate the true benefit. Quality of life and health status questionnaires, including EORTC QLQ-C30, EORTC QLQ-BILI, and EQ-5D, were collected in ABC-06, but the results have yet to be reported [37]. EORTC QLQ-C30 was also collected in the NIFTY trial over the course of 8 cycles of treatment [38]. Presented quality of life data from NIFTY is suggestive that there is no significant difference in HRQoL between patients treated with nal-IRI plus 5-FU/LV compared to 5-FU/LV alone.

Chemotherapy-induced peripheral neuropathy (CIPN) is often a major concern for both clinicians and patients as it has the potential to significantly impact HRQoL. While platinum agents are frequently associated with CIPN, the incidence and severity of CIPN varies depending on the choice of chemotherapy agent, dose, and duration of treatment. Notably, the reported incidence of grade 3 or greater CIPN in clinical trials utilizing platinum agents in treating advanced BTC are low [31,32,33,37]. Regardless, clinicians are advised to assess for neuropathy throughout the course of treatment and consider dose delays, dose reductions or discontinuation in those patients who develop intolerable symptoms or functional impairment [39]. To date, duloxetine is the sole agent with convincing data supporting its use in existing CIPN [40,41]. However, there is great interest in identifying new preventative and treatment strategies for CIPN.

Prognostic factors can play a vital role in identifying patients who would benefit from chemotherapy to prolong survival, alleviate tumor-related symptoms, and improve quality of life. ECOG performance status, disease status, number of metastatic sites, including presence of liver metastasis, gender, bilirubin, white blood cell count, neutrophil count, neutrophil-lymphocyte ratio, and hemoglobin level, have been identified as possible independent prognostic factors for BTC patients treated with chemotherapy [42,43,44,45]. Unfortunately, to date, these factors have limited accuracy in determining clinical outcomes.

## 4. Immunotherapy: Emerging Role in Advanced BTC and Known Adverse Effects

Immune checkpoint inhibitors (ICIs) have radically changed the treatment paradigm for several solid malignancies. However, the role of ICIs in management of BTCs has yet to be clearly established. The early phase IB KEYNOTE-028 basket study of patients with select PD-L1 positive advanced or metastatic tumors, including BTC, suggested pembrolizumab had promising anti-tumor activity, with an overall response rate (ORR) of 13.0% [46,47]. The subsequent KEYNOTE-158, a nonrandomized, open-label, phase II trial, went on to evaluate pembrolizumab monotherapy in patients with advanced BTC, regardless of PD-L1 status, but was unable to replicate the same anti-tumor activity, instead reporting an ORR of 5.8%, a median PFS of 2.0 months, and a median OS of 7.4 months [46]. Neither study included specific information on primary tumor location.

Despite these initial disappointing results, several trials are ongoing assessing the role of ICI monotherapy and in combination with other systemic anticancer therapies [47,48]. A recently published multicenter, phase II trial nivolumab has modest efficacy in patients with advanced refractory cholangiocarcinoma and gallbladder cancer, with an objective response rate of 11–22% [49]. However, approximately 40% of responders in this trial demonstrated durable objective response lasting at least one year. Meanwhile, a multicenter, open-label, phase I trial completed in Japan assessing patients treated with nivolumab compared to nivolumab with gemcitabine plus cisplatin reported a median OS of 5.2 months and 15.4 months, respectively [50]. The majority of patients included in this trial had microsatellite-stable tumors and either iCCA or GBC. Given these unprecedented findings, there is great interest in exploring other immune-based combinations and several eagerly anticipated studies are underway [51,52,53].

Overall, ICIs generally well tolerated both as monotherapy, in combination and in conjunction with other systemic therapies. Safety data from KEYNOTE-028 and KEYNOTE-158 noted most adverse events were mild-to-moderate with few experiencing grade 3 toxicities, 16.7% and 12.5%, respectively [46]. In fact, neither trial reported grade 4 treatment-related adverse effects. Immune-mediated adverse events of any grade occurred in 16.7% to 18.3% of patients treated with pembrolizumab. In comparison, frequency of grade 3 or 4 toxicities and immune-mediated adverse events were higher in the recent phase II trial with nivolumab, 17% and 52%, respectively [49].

Predictably, in the phase I trial of nivolumab with or without gemcitabine plus cisplatin, combination chemoimmunotherapy was more frequently associated with grade 3–4 treatment-related adverse events compared to nivolumab alone (90% versus 10%). While immune-mediated adverse events were seen in both arms, patients treated with combined therapy reported several toxicities more specific to chemotherapy, including peripheral neuropathy, alopecia, and low cell counts. The immune-mediated toxicities of ICIs are well documented, and their management is outlined in multiple national and international guidelines, thus not being further detailed in this review [54,55,56].

## 5. Targeted Therapies: Toxicities and Management Strategies

Molecular profiling has found BTCs are target-rich malignancies, with upwards of 83% of tumors having clinically relevant or potentially actionable genetic alterations [57,58,59,60]. Specific patterns of genetic alterations have been observed for anatomic sub-types of BTC. Isocitrate dehydrogensase (*IDH*) and fibroblast growth factor receptor (*FGFR*) mutations tend to cluster in iCCA, while HER2 (*ERBB2*) alterations are more frequent in eCCA and GBC [61,62]. Alterations in neurotropic tyrosine kinase receptor *NTRK* and *BRAF* have also been noted in BTCs, regardless of anatomic sub-type, although their prevalence is typically less than 5% [12,61,62,63]. Currently, multiple targeted therapies for advanced BTC are being evaluated in clinical trials both as monotherapy and in conjunction with chemotherapy. As targeted therapies are incorporated more into clinical practice, it is vital to recognize the specific side effects associated with each.

### 5.1. FGFR Tyrosine Kinase Inhibitors

FGFRs regulate a wide range of biological functions in cells, including proliferation, survival, migration, and differentiation. FGFRs 1–4 are transmembrane receptors containing an extracellular ligand binding domain, as well as an intracellular tyrosine kinase domain. FGFR fusions are present in approximately 20% of patients with iCCA, with *FGFR2* fusions being the most common aberration [61,62,63]. Among the FGFR tyrosine kinase inhibitors (TKIs) that have been developed to date, pemigatinib, infigratinib, and futibatinib have shown promising results in clinical trials [64,65,66,67,68].

FIGHT-202, a multicenter, open-label, single-arm, phase II trial evaluated the safety and anti-tumor activity of pemigatinib in patients with locally advanced or metastatic CCA and disease progression following prior systemic therapy [64]. The majority of patients enrolled in FIGHT-202 carried a diagnosis of iCCA with an FGFR2 fusion or rearrangement. Of the patients with FGFR2 alterations, 35.5% achieved an objective response. Median PFS in this population was 6.9 months, and, while the data was not mature at the data cutoff, median OS was estimated at 21.1 months. Notably, patients with other FGFR alterations did not achieve a measurable response with a median PFS of 2.1 months and median OS of 6.7 months.

Similarly, the mature results from a multicenter, open-label, single-arm, phase II study recently published in the Lancet Gastroenterology and Hepatology suggest that infigratinib has promising activity in patients with advanced or metastatic CCA [65] and progression after at least one other prior systemic therapy. Patients with GBC and AVC were specifically excluded. Interestingly, more than half of the patients enrolled had received two or more previous lines of therapy. ORR was estimated at 23.1% for patients with an FGFR2 alteration, with further subgroup analysis finding objective response rate numerically varied, depending on the number of previous lines of therapy. Despite the majority of the study population being heavily pre-treated, median PFS was 7.3 months, and median OS was 12.2 months.

Futibatinib has also been shown to have meaningful clinical benefit in previously treated patients with advanced of metastatic iCCA. FOENIX-101, a phase I clinical trial, found futibatinib not only had a tolerable safety profile but also encouraging activity in this population with most responses occurring within 3 months and lasting more than 6 months [66]. FOENIX-CCA2, a global open-label phase II study assessing the efficacy of futibatinib in this same population is ongoing. However, interim analyses presented recently at the 2020 European Society for Medical Oncology (ESMO) Virtual Congress and the ASCO 2020 Annual Meeting found an objective response rate of 37.3% and a median PFS of 7.2 months [67,68,69,70].

FGFR inhibitors are known to be associated with side effects common to most TKIs, such as diarrhea and vomiting. Furthermore, depending on the breadth of their inhibitory targets, FGFR TKIs may also have adverse effects related to inhibition of vascular endothelial growth factor receptor (VEGFR), resulting in hypertension, cardiovascular events, and proteinuria. However, specific inhibition of FGFR signaling can lead to unique adverse events, including hyperphosphatemia, dermatologic toxicities, and ocular toxicities (Table 1).

Hyperphosphatemia is believed to result from the alteration of vitamin D and phosphorus metabolism which is mediated via FGF23 [77]. In clinical trial, hyperphosphatemia of any grade occurred in 59–77% of patients treated with an FGFR TKI [64,65,66]. Only a small percentage of patients, approximately 12–13%, experienced grade 3 to 4 hyperphosphatemia, which has been attributed to early and aggressive management. Typically, hyperphosphatemia occurred early after treatment initiation, with one study reporting a median time to onset of 15 days [64]. Depending on the toxicity grade, hyperphosphatemia was then managed with low-phosphate diet, concomitant phosphate binders, diuretics, dose reduction, and even drug interruption.

In clinical practice, it is advised that all patients should be educated on dietary modifications that can reduce the risk of developing hyperphosphatemia when initiating FGFR inhibitors. It is critical to note that dietary modifications are an important first step in management but may not maintain phosphorous levels within a normal range. Otherwise, current management recommendations reflect what has been done in trials. Specifically, clinicians should consider starting phosphate binders once serum phosphate levels are greater than 5.5 mg/dL. Clinicians should then reduce the dose or interrupt treatment if levels are greater than 7 mg/dL on two separate occasions or greater than 10 mg/dL, despite optimizing diet and phosphate binders [64,78]. Ultimately, permanent discontinuation should be considered if phosphate levels remain greater than 10 mg/dL, despite two dose reductions and/or drug interruption (Table 2).

Common dermatologic toxicities associated with FGFR inhibitors reported in clinical trials include nail bed infections, onycholysis, hand-foot syndrome (HFS), alopecia, mucositis, stomatitis, xerostomia, and calciphylaxis [64,65,66]. In the phase II FIGHT-202 trial of pemigatinib, dermatologic toxicity led to dose reduction or interruption in 3% and 7% of patients, respectively. Among dermatologic toxicities of any grade, stomatitis (20–54%) and alopecia (14–46%) of any grade are reported most commonly. While stomatitis developed rapidly after treatment initiation, alopecia and nail changes were typically noted after a few months.

Dermatologic toxicities can adversely impact quality of life, so prevention and early management are key (Table 2). All patients should be educated on anticipated dermatologic toxicities and preventative strategies when starting therapy. Some important preventative strategies include good oral hygiene, regular dentist visits, and use of topical emollients, as well as avoidance of prolonged contact with water, repeated trauma, and pressure [79]. Patients should also be instructed to seek medical evaluation for onycholysis and stomatitis to rule out concomitant infection. In grade 3 or greater dermatologic toxicities that do not improve with standard topical treatments, early dermatology referral is warranted, and an FGFR inhibitor should be withheld [79]. Once symptoms improve to grade 1 or less, the drug can be restarted at a lower dose. FGFR inhibitors should be permanently discontinued if the patient has persistent grade 3 toxicity, despite two dose reductions, in the event of a grade 4 or 5 side effect, or if the patient develops calcinosis cutis/calciphylaxis.

Finally, FGFR inhibitors are also associated with unique ocular toxicities. Dry eye is the most frequently reported ocular adverse event of any grade for pemigatinib (21%), infigratinib (38%), and futibatinib (11%) [64,65,66]. Central serous retinopathy and retinal detachment, which can lead to permanent vision loss if not treated, were also reported as adverse events in clinical trials with FGFR inhibitors. FGF plays an integral role in maintaining the integrity of retinal pigment epithelium, so it is not surprising retinal damage may result from FGFR inhibition [80]. While less common than dry eye, serous retinopathy and/or retinal detachment were reported in 4% of patients treated with pemigatinib, 3% treated with futibatinib, and 17% treated with infigratinib [64,65,66].

Ophthalmology consultation is recommended prior to initiating FGFR inhibitors and for all patients reporting vision changes. As serous retinopathy and retinal detachment are reversible, early identification, ophthalmologic evaluation, and discontinuation of FGFR inhibitor are important for patients experiencing grade 3 or greater toxicity. FGFR inhibitor can be restarted at a dose reduction under close supervision of an ophthalmologist in patients who have resolution of symptoms after 4 weeks [78].

Quality of life is an exploratory or secondary outcome in several ongoing clinical trials with FGFR inhibitors [81,82,83]. While limited, the HRQoL data that is available suggests that FGFR inhibitors do not adversely impact quality of life. A longitudinal evaluation of quality of life in patients treated with pemigatinib as part of the FIGHT-202 is suggestive that quality of life was impacted more by progression of disease than the treatment itself [84]. Similarly, HRQoL data from the phase II FOENIX-CCA2 trial shows that physical, cognitive, and emotional functioning were maintained in patients treated with futibatinib [85].

### 5.2. IDH Inhibitors

IDH mutations are found in approximately 20% of iCCA but are otherwise seen rarely in GBC and eCCA [12,61,62]. IDH mutations are prevalent in several other rare malignancies, such as glioma, acute myeloid leukemia, and thyroid carcinoma, so that multiple IDH-selective inhibitors have been developed [86]. At this time, Ivosidenib is the most developed IDH inhibitor for CCA.

In the phase III ClarIDHy clinical trial, median PFS was significantly improved in patients with *IDH1*-mutant advanced chemotherapy-refractory iCCA treated with Ivosidenib compared to placebo [71]. Treatment-related adverse events leading to Ivosidenib being discontinued occurred in 2% of patients, and treatment-emergent adverse events requiring a dose reduction occurred in 3% of patients. Quality of life assessments using the EORTC QLQ-C30 and the QLQ-BIL21 were also completed at baseline, every 12 weeks, until the start of a new anticancer therapy and at the safety follow up visit. While a decline in physical functioning from baseline to cycle 2 day 1 was noted in both Ivosidenib and placebo arms, the decline was significantly less for those in the Ivosidenib group.

Despite being well tolerated, Ivosidenib is not without potential side effects (Table 1). Reported adverse events for Ivosidenib in solid tumors are far fewer and more manageable compared to those reported in hematologic cancers [87]. The most frequently reported adverse events of any grade include fatigue (12–23%), nausea (20–33%), diarrhea (11–31%), vomiting (10–17%), and prolonged QT interval (8–11%) [71,87,88]. Given the risk of QT prolongation, attempts should be made to avoid concomitant medications associated with QT prolongation in patients treated with Ivosidenib. Clinicians should also monitor electrolytes and electrocardiograms (ECGs) during the course of treatment, particularly in patients with nausea, vomiting, or diarrhea (Table 2).

### 5.3. HER2 Targeted Therapies

HER2/neu overexpression is found in approximately 5–15% of BTCs, most being GBC or eCCA [12,61,62]. Several phase II trials are currently investigating HER2-directed therapies in BTC, including the TAPUR (NCT02693535) and MATCH (NCT02465060) trials. However, therapies targeting HER2 have demonstrated modest activity so far in BTC. The phase IIa MyPathway basket study reported an objective response rate of 23% with pertuzumab plus trastuzumab [72]. Of the 39 patients included, 16 (41%) had primary GBC, 7 (18%) had eCCA, 7 (18%) had iCCA, and 5 (13%) had AVC. Treatment-related adverse events occurred in 62% of patients, most commonly diarrhea (26%), elevated transaminases (10%), and infusion-related reaction (10%).

A phase II trial of lapatinib, a dual epidermal growth factor receptor (EGFR) and HER2/neu, failed to show activity as a single agent in patients with advanced BTC. Of note, primary tumor site was not differentiated within the publication materials. Overall the reported toxicity profile lapatinib was similar to that of pertuzumab plus trastuzumab [73]. Interestingly, while cardio-toxicity is a well recognized adverse effect of HER2-targeted therapies, adverse cardiac events were not reported in patients with BTC treated with pertuzumab plus trastuzumab or lapatinib in clinical trial [72,73,89].

To date, quality of life data has not been published for patients with advanced BTC treated with HER2 directed therapies. This is may be in part due to the fact that the majority of studies on HER2-directed therapies in this population are basket trials. In advanced gastric cancer, at least trastuzumab in combination with chemotherapy did not worsen and actually preserved HRQoL longer compared to chemotherapy alone [90]. Further investigation into the effect of HER2-directed therapies on survival and quality of life is warranted in patients with advanced BTC.

### 5.4. TRK Inhibitors

NTRK fusions are identified in 4% of patients with BTC. Currently approved first-generation tropomyosin receptor kinase (TRK) inhibitors larotrectinib and entrectinib are not only active against NTRK fusions but also ROS1 and ALK fusions, which are reported in upwards of 8.7% and 2.7% of patients with BTC, respectively [58,62,91]. Pooled analyses of phase 1 or 2 trials of entrectinib and larotrectinib found, respectively, 4% and 2% of patients discontinued treatment due to a drug-related adverse event [74,75]. Furthermore, adverse events associated with either drug were low and primarily grade 1 or 2. The most common adverse events of any grade included elevation of transaminases, fatigue, nausea, vomiting, dizziness, diarrhea, constipation, and cough (Table 1).

The TRK signaling pathway plays a role in nervous system development and maintenance. Predictably, on-target adverse events associated with entrectinib and larotrectinib include dizziness, paraesthesias, weight gain, and cognitive changes [92]. Frequency of these neurologic on-target adverse events range from 16–25%, with the majority being grade 1 or 2 events. A single patient was reported to have a dose-limiting grade 3 cognitive disturbance with entrectinib but was treated at a dose above the recommended phase II dose [74,75,92]. Consequently, unless a grade 3 or 4 toxicity, the development of one of these neurologic adverse events does not necessarily require dose reduction nor drug interruption [93].

TRK inhibitors have not only favorable safety profiles and high response rates but also favorable impact on quality of life. An expanded quality of life analysis of patients treated with larotrectinib found most patients had clinically meaningful quality of life improvements [94]. Patient reported outcomes from the phase II basket study STARTRK-2 similarly found the adverse events related to entrectinib had minimal impact on patients’ HRQoL [95].

### 5.5. BRAF/MEK Inhibitors

BRAF mutations are reported in approximately 5% of BTC [61,62,63]. In the phase II ROAR basket trial, dual targeting with dabrafenib, a BRAF inhibitor, and trametinib, an MEK inhibitor shows promising activity in patients with *BRAF* V600E mutated locally advanced, metastatic or recurrent CCA or GBC with an ORR of 42% [76]. Over 90% of the patients enrolled carried a diagnosis of iCCA. Dose reduction or interruption due to treatment-related adverse event was needed for 35% and 56% of patients, respectively. Serious treatment-related adverse events occurred in 21% of patients, the most frequent of which was pyrexia (19%). Quality of life data in this population is not available at this time.

Pyrexia has been observed in patients receiving dabrafenib monotherapy and may be accompanied by rigors, dehydration, hypotension, weakness, or dizziness. The etiology of dabrafenib-induced pyrexia remains unclear. Typically, pyrexia occurs within the first four weeks of starting treatment. Patients should be counseled on the importance of reporting febrile episodes while on dabrafenib and trametinib (Table 2). In addition to infectious workup and anti-pyretic treatments, dabrafenib should be held until pyrexia resolves, at which time it can be restarted at the same dose or with a dose reduction if other symptoms, such as hypotension, were present. A concomitant course of oral corticosteroids consisting of prednisone 10 mg for five days can be considered in patients with subsequent febrile events [76,96].

## 6. The Role of Supportive Oncology and Palliative Care Services

Early integration of palliative care or supportive oncology services should be considered for patients with advanced BTC. Historically, palliative care has been primarily offered to patients approaching the end of life. However, palliative or supportive care encompasses more than just end of life symptom management. Key domains managed by quality palliative care include the social, spiritual, cultural, and psychologic and psychiatric aspects of care [97]. Consequently, palliative care can be complementary to oncology care in ensuring patients understand their illness and have their symptoms and goals of care addressed.

Across multiple large clinical trials, early palliative care was associated with higher HRQoL and lower rates of depression when compared to standard of care in patients with advanced cancers [98,99,100]. Notably, the effectiveness of early palliative care has been primarily studied in mixed populations, without separation of results based on cancer type. Temel and colleagues specifically assessed the impact of early integrated palliative care in patients with newly diagnosed gastrointestinal (GI) and lung cancers, including a subgroup analysis by cancer type [101]. While their study confirmed early integrated palliative care was associated with better HRQoL and mood, the subgroup analysis of the GI found no difference in HRQoL between intervention arms. The significance of this finding in GI malignancies is unclear as the sample sizes by sub-type were small but highlights the need for further research on the impact of palliative care by cancer type.

The guidelines provided by the WHO and ASCO recommend dedicated palliative or supportive care services early in the course of disease, concurrent with active treatment for patients with advanced cancers [102,103]. Yet, how to best integrate oncology and palliative care still has to be established [104]. Hospital-at-home programs have been proposed as a model for integrating palliative care that would address patients’ needs at home or provide alternatives to emergency department (ED) use and hospitalization. Mooney and colleagues conducted a prospective, nonrandomized, real-world cohort comparison of patients managed an oncology hospital at home program or standard of care [105]. The hospital at home intervention was associated with fewer unplanned hospitalizations, shorter hospital length of stay, and fewer ED visits. Regardless of the model of palliative care available, clinicians should not hesitate to involve supportive oncology services early in the course of disease for patients with advanced BTC.

## 7. Conclusions

Over the last decade, the development of novel targeted therapies and new combinations of chemotherapy have inspired hope for more treatment options for advanced BTC. Despite the many advances in the field, unresectable and metastatic BTC continues to have an overall dismal prognosis. Complicating management, patients frequently suffer from poor HRQoL. In these patients, the prevention and easing of suffering due to tumor- and treatment-related symptoms is of primary importance. It is imperative that clinicians recognize complications from advanced BTC and its systemic treatment and manage them properly. Furthermore, clinical trials in advanced BTC should continue to assess how future treatments impact quality of life, and palliative care/supportive oncology should be an integral part of patients’ care.

## Figures and Tables

**Table 1:** Treatment-related adverse events of any grade observed in clinical trials of targeted therapies in advanced BTC.

Drug	Target	Study	Diarrhea	Fatigue	Nausea	Pyrexia	Hyperphosphatemia	Skin Toxicity	Ocular Toxicity	CNS Toxicity
Pemigatinib	FGFR 1-3 VEGFR2	Phase II trial FIGHT-202 [64]	36%	42%	25%	-	55%	Stomatitis: 32% Alopecia: 46% Xerostomia: 29% HFS: 15% Onycholysis: 5%	Dry eye: 25% Corneal/retinal disorders: 2%	-
Infigratinib	FGFR 1-3/4	Phase II trial PROOF 301 [65]	18%	29%	10%	1%	74%	Stomatitis: 51% Alopecia: 32% Xerostomia: 21% HFS: 32% Onycholysis: 12%	Dry eye: 31% Corneal/retinal disorders: 17%	Headache: 17%
Futibatinib	FGFR 1-4	Phase I trial FOENIX-101 [66]	34%	16%	30%	11%	68%	Stomatitis: 20% Alopecia: 14% Xerostomia: 25% Onycholysis: 2%	Dry eye: 11% Corneal/retinal disorders: 3%	Headache: 5%
Ivosidenib	IDH1	Phase III trial ClarIDHy [71]	29%	24%	32%	10%	-	Rash: 7%	-	Headache: 9% Neuropathy: 5%
Pertuzumab/ Trastuzumab	HER2/neu	Phase IIa trial MyPathway [72]	33%	18%	15%	15%	-	Rash: 13%	-	-
Lapatinib	EGFR HER2/neu	Phase II trial [73]	67%	78%	78%	-	-	Rash: 33%	-	-
Larotrectinib	TRK	Phase I/II pooled analysis [74]	24%	32%	25%	20%	-	Rash: 6%	Blurred vision: 3%	Dizziness: 25% Headache: 15% Paraesthesia: 7% Cognitive disorder: 1%
Entrectinib	TRK ROS1 ALK	Phase I/II pooled analysis [75]	34%	38%	34%	21%	-	Rash: 7% Dry skin: 7%	Blurred vision: 9%	Dizziness: 35% Paraesthesia: 18% Cognitive disorder: 8%
Dabrafenib/ Trametinib	BRAF/MEK	Phase II trial ROAR [76]	30%	33%	42%	67%	-	Rash: 25% Xerostomia: 19% Eczema: 12%	-	Headache: 23%

**Table 2:** Prevention and management of key adverse events related to targeted therapy use.

Adverse Event	Prevention	Management Strategies
Hand-foot syndrome	-Prophylactic removal of hyperkeratotic areas -Soft socks and comfortable shoes -Avoid activities causing repeated trauma or pressure to hands and feet -Limit exposure to harsh chemicals and heat	-Topical moisturizing cream containing urea 10% -Consider high potency topical steroids for grade >2 symptoms
Stomatitis	-Complete dental work to eliminate tooth and gum disease prior to start of treatment -Thorough and frequent cleaning of the oral cavity -Avoid salty, spicy, or citrus-based foods -Use of ice chips	-Evaluation to rule out concomitant infection -Anti-microbial prophylaxis or treatment as clinically indicated -Dexamethasone or sucralfate containing mouth washes
Nail toxicity	-Avoid prolonged contact with water, repeated trauma, friction, and pressure on nails and nail bed -Avoid biting nails or cutting nails too short -Protective gloves -Limiting use of nail polish removers and nail hardeners	-Evaluation to rule out concomitant infection -Anti-microbial treatment as clinically indicated -Topical povidone iodine twice daily -Daily vinegar and water (1:1) soaks
Ocular toxicity	-Ophthalmic exam at baseline, as well as 4–6 weeks after starting an FGFR inhibitor	-Artificial tears for dry eye -Immediate ophthalmology consult for vision changes
Hyperphosphatemia	-Provide education on dietary modifications to eliminate foods high in phosphate -Close monitoring of serum phosphate levels	-Consider phosphate binder once phosphate >5.5 mg/dL -Start phosphate binder and consider dose reduction or interruption if phosphate >7 mg/dL on two separate checks or >10 mg/dL, despite optimization of diet and phosphate binders -Consider permanent discontinuation if phosphate >10 mg/dL, despite two dose reductions
Prolonged QT	-Avoid concomitant QT prolonging medications when starting an IDH1 inhibitor -Monitor and supplement electrolyte levels as indicated during the course of treatment, particularly in patients with nausea, vomiting, or diarrhea	-QTc 480–500 msec requires dose reduction but may increase to the prior dose after 2 weeks if QTc returns to <480 msec -QTc >500 msec on two separate checks requires dose interruption but may be restarted with dose reduction after 2 weeks if QTc returns to <480 msec
Pyrexia	-Educate patients to report febrile episodes -Consider secondary prophylaxis with anti-pyretic treatments in patients with prior severe febrile reactions fever with complications	-Evaluation to rule out infectious etiology -Anti-pyretic treatments -Dabrafenib-induced pyrexia: -Hold dabrafenib until afebrile -Restart at the same or reduced dose -Consider course of steroids for 5 days in patients with fever lasting >3 days or subsequent febrile events
CNS toxicity	-Educate patients regarding potential CNS toxicity, particularly if starting TRK inhibitor therapy	-Consider dose interruption, followed by dose reduction in grade >3 toxicity and in patients with intolerable grade 2 symptoms unresponsive to pharmacologic management -Consider discontinuing treatment for grade >4 toxicity -Meclizine or scopolamine for ataxia or vertigo -Adequate fluid resuscitation for orthostasis -Consider midodrine, fludrocortisone, or droxidopa for orthostasis
